# Current advances in understanding the molecular profile of hereditary diffuse gastric cancer and its clinical implications

**DOI:** 10.1186/s13046-023-02622-3

**Published:** 2023-03-04

**Authors:** Hui Jun Lim, Lizhe Zhuang, Rebecca C. Fitzgerald

**Affiliations:** 1 Department of Oncology, Early Cancer Institute, University of Cambridge, Box 197, Cambridge Biomedical Campus, CB2 0XZ Cambridge, UK; 2 Department of Sarcoma, Peritoneal and Rare Tumors (SPRinT), Division of Surgery and Surgical Oncology, National Cancer Centre Singapore, Singapore, Singapore

**Keywords:** Hereditary diffuse gastric cancer, Signet ring carcinoma, *CDH1* pathogenic variant, Second-hit mechanism, Synthetic lethality

## Abstract

Hereditary diffuse gastric cancer (HDGC) is an autosomal dominant cancer syndrome attributed to germline *CDH1* mutations that carries a high risk for early onset DGC. HDGC raises a significant health issue due to its high penetrance and mortality unless diagnosed early. The definitive treatment is to undergo prophylactic total gastrectomy which is associated with significant morbidity., highlighting the urgent need for alternative treatment methods. However, there is limited literature examining potential therapeutic strategies building on emerging insights into the molecular basis of progressive lesions in the context of HDGC. The aim of this review is to summarise the current understanding of HDGC in the context of *CDH1* pathogenic variants followed by a review of the proposed mechanisms for progression. In addition, we discuss the development of novel therapeutic approaches and highlight pertinent areas for further research. A literature search was therefore performed for relevant studies examining *CDH1* germline variants, second-hit mechanisms of *CDH1*, pathogenesis of HDGC and potential therapeutic strategies in databases, including PubMed, ScienceDirect and Scopus. Germline mutations are mostly truncating *CDH1* variants affecting extracellular domains of E-cadherin, generally due to frameshift, single nucleotide variants or splice site mutations. A second somatic hit of *CDH1* most commonly occurs via promoter methylation as shown in 3 studies, but studies are limited with a small sample size. The multi-focal development of indolent lesions in HDGC provide a unique opportunity to understand genetic events that drive the transition to the invasive phenotype. To date, a few signalling pathways have been shown to facilitate the progression of HDGC, including Notch and Wnt. In in-vitro studies, the ability to inhibit Notch signalling was lost in cells transfected with mutant forms of E-cadherin, and increased Notch-1 activity correlated with apoptosis resistance. Furthermore, in patient samples, overexpression of Wnt-2 was associated with cytoplasmic and nuclear β-catenin accumulation and increased metastatic potential. As loss-of-function mutations are challenging to target therapeutically, these findings pave the way towards a synthetic lethal approach in *CDH1*-deficient cells with some promising results in-vitro. In future, if we could better understand the molecular vulnerabilities in HDGC, there may be opportunities to offer alternative treatment pathways to avoid gastrectomy.

## Background

Hereditary diffuse gastric cancer (HDGC) is an autosomal dominant cancer syndrome attributed to germline mutations within *CDH1* which is responsible for up to 3% of all gastric cancers [1, 2]. *CDH1* maps to chromosome 16q22.1 and consists of 16 exons which encode the cell-to-cell adhesion protein called E-cadherin [3]. E-cadherin is a transmembrane glycoprotein located at adherens junctions in epithelial tissues with cell-cell adhesion and signal transduction functions [4]. It has 3 structural domains, namely extracellular, transmembrane and cytoplasmic [5]. The cytoplasmic domain connects to the actin cytoskeleton through various catenins and regulates basic cellular processes, including cell signalling, apoptosis and invasion [6]. It plays a key part in carcinogenesis where its role in tumour progression and epithelial-mesenchymal transition (EMT) has been widely studied in various malignancies [7, 8]. Loss of the second allele of E-cadherin deficiency has been reported to initiate carcinogenesis in HDGC [9, 10].

The HDGC syndrome was first described in three extended New Zealand Māori families in 1998 [11]. Although HDGC is uncommon, it raises a significant health issue due to its high penetrance, early age at presentation and propensity for a late diagnosis. It is dominated by risk for early onset of DGC in conjunction with an elevated risk of lobular breast cancer [12]. Notably, affected patients have a cumulative risk of gastric cancer of 56 and 70% by the age of 80 years for females and males respectively [13]. As such, the current clinical guideline for individuals with a germline mutation in *CDH1* is to undergo prophylactic total gastrectomy (PTG) in early adulthood between 20 to 30 years of age [14]. However, this procedure is associated with significant morbidity which can have an adverse impact on the quality of life of patients [15, 16].

The multi-focal development of indolent and early invasive lesions in HDGC provides a unique opportunity to understand genetic events that drive the transition to the invasive phenotype [17, 18]. Through greater insight of disease pathogenesis, this may facilitate risk stratification of patients to guide management and follow-up. In addition, this will be a valuable opportunity to identify potential therapeutic targets such that HDGC may be prevented by early interception in at-risk individuals, ultimately obviating the need for PTG. Hence, an increased understanding of the mechanisms driving the transition through the different stages of HDGC progression would direct the development of risk stratification and targeted treatment options for HDGC patients.

In this literature review, the current understanding of HDGC in the context of *CDH1* pathogenic variants will be discussed in conjunction with recent advances in molecular processes underlying crucial steps of DGC development. In addition, we will discuss their utility in the development of novel therapeutic approaches. Lastly, pertinent areas for further research in HDGC will be examined with a translational approach.

## Pathogenic germline variants in *CDH1*

CDH1 germline variants refer to loss of function mutations in either of the two alleles. However, these cells with CDH1 germline variants are not regarded as malignant as the other allele is able to retain normal function. Since the first germline mutation of *CDH1* was reported in families in indigenous New Zealand Māori in 1998, greater than 100 germline mutations have been found distributed evenly throughout the exons [19–21]. Although no major mutational hotspots have been identified, reports on the same pathogenic variant have been found in unrelated families, including c.1003C > T, 1137G > A and c.1901C > T in exon 7, 8 and 12 respectively [22]. Germline mutations are mostly truncating *CDH1* variants affecting extracellular domains of E-cadherin, generally due to frameshift, single nucleotide variants or splice site mutations [20, 21, 23]. In comparison, missense mutations and structural variants, including large re-arrangements and large deletions involving multiple exons of the gene, occur less commonly and account for 28 and 5% of mutations respectively [22, 24, 25]. Notably, most truncating mutations in *CDH1* are pathogenic [26, 27]. A study of 152 HDGC families reported those with truncating *CDH1* germline variants in the PRE-PRO region were 6 times more likely to have family members affected by colorectal cancer compared with germline variants in other regions of the gene [28]. In contrast, the functional and clinical consequence of missense mutations are less predictable as the effect of the sequence variants on E-cadherin function may vary [29, 30]. A full-length protein is preserved most of the time with normal E-cadherin expression for missense mutations [31]. To aid in characterisation of these mutations, a group from Portugal has developed an in vitro assay to quantify and map E-cadherin expression for *CDH1* germline variants by combining Western-blotting, immunocytochemistry and bio-imaging techniques [29]. Furthermore, to elucidate defects in the epithelial structure and morphology that can arise from E-cadherin mutants, the group developed a platform that identifies and quantifies cellular distribution patterns using in situ-microscopy images [29]. Nonetheless, no definite correlation between the nature of germline mutation and phenotype has been established

For families without recognised inactivating *CDH1* mutations, they may have mutations in regulatory sequences or germline mutations in other relevant genes that contribute to the same pathway [32, 33]. An examination of more than 150,000 individuals with breast or gastric cancer revealed that 0.02% had a *CTNNA1* loss-of-function mutation and of those 12% had DGC [34]. *CTNNA1* encodes the protein α-E-catenin which functions in a complex with β-catenin where it binds the cytoplasmic domain of E-cadherin to the cytoskeleton [35]. As *CTNNA1* is involved in intra-cellular adhesion, this loss-of-function mutation is likely similar to that of *CDH1* mutations, although disease penetrance remains to be defined [36]. There are also mutations in genes associated with other cancer-predisposition syndromes, such as *BRCA2* generally associated with hereditary breast and ovarian cancer [37], hence in some families with *BRCA2* mutations, an increased incidence of gastric cancer has been encountered with one family fulfilling the HDGC criteria [38]. Novel germline mutations in other genes have also been reported, including *MAP 3 K6*, *STK11*, *PRSS1*, *MSR1* and *PALB2* [39–41]. For example, germline loss-of-function mutations in *PALB2* were more than 7.5 times more common in HDGC families compared to the general population [41]. The most recent international guidelines recommend *PALB2* testing to be considered in unexplained families alongside other genes associated with an increased risk of gastric cancer, such as *BRCA2*, *APC* and *TP53* [14]. Nonetheless, the clinical implications of these genes remain to be determined.

## Second-hit mechanism of *CDH1* wild-type allele

The second hit of *CDH1* refers to loss of function of the wild-type allele which leads to complete dysfunction of *CDH1* with consequential effects on various signalling pathways that drastically alter the cells’ behaviour. Together, the *CDH1* germline variant and second hit of the wild-type allele form the molecular basis of HDGC. Epigenetic alterations are suggested to play an essential role in this process where the most common mechanism leading to biallelic *CDH1* inactivation is promoter hypermethylation and less frequently, somatic mutation or loss of heterozygosity (LOH) [42, 43]. In the literature, there are 4 studies so far which have evaluated the second-hit mechanism of *CDH1* in HDGC [44–47]. The largest study was from Portugal which quantified the different second hit mechanisms in *CDH1* in neoplastic lesions from 17 HDGC patients among 15 families comprising of 16 primary tumours and 12 metastases [44]. The study showed somatic *CDH1* epigenetic and genetic alterations were detected in lesions from 80% of HDGC families and in 75% of all lesions analysed. Of the 28 neoplastic lesions analysed, 32.1, 25.0 and 17.9% was found to have promoter hypermethylation, LOH or both alterations respectively. Furthermore, 50% of *CDH1* second hits in primary tumours were epigenetic modifications while a significantly greater proportion in metastases were LOH at 58.3%. Similarly, an additional study evaluated possible second-hit mechanisms of *CDH1* whereby E-cadherin promoter methylation status and loss of heterozygosity in 16 patients with *CDH1* germline mutations were examined [45]. Notably, 6 patients had at least 1 sec hit mechanism in which 2 exonic mutations consisting of truncating and missense mutations, and 4 intronic mutations which affecting splicing were identified. Furthermore, tumours from 4 patients had promoter hypermethylation. E-cadherin loss correlated with identification of a second hit. This study demonstrated inactivation of the second *CDH1* allele occurred by mutation and methylation events. Similarly, an additional study showed that a *CDH1* intragenic deletion was the second hit inactivating the wild-type allele in one of 4 family members with HDGC [46], while another study reported 3 out of 6 patients had aberrant *CDH1* promoter methylation [47]. Overall, these studies showed the second hit in *CDH1* most commonly occurs via promoter methylation in HDGC.

## Proposed models of pathogenesis in HDGC

With an inherited germline *CDH1* mutation coupled with inactivation of the wild-type allele, HDGC is likely a multi-stage process with initial loss of E-cadherin and disruption of apical-basal cell polarity which enables tumour cells to detach from the basement membrane [48]. It typically presents with a unique early stage (T1a) characterised by foci of intra-mucosal signet ring cells (SRC) confined to the lamina propria [49]. While the natural history of these early intra-mucosal lesions is not completely understood, it is suggested that they remain indolent with a prolonged latency period before sub-mucosal invasion and metastasis [49]. A key challenge is to distinguish between indolent lesions and those at high risk for cancer progression. By comparing the genomic and phenotypic features of indolent and invasive lesions, the hope is that this will facilitate risk stratification of patients to guide management and follow-up. In addition, this will be a valuable opportunity to uncover novel therapeutic targets for early interception in at-risk individuals. The complete loss of *CDH1* expression as a single genetic change is not sufficient for the development of invasive carcinoma as shown in animal models where conditional knockout of *CDH1* in mice induced SRC but not the development of carcinoma invading into submucosa [50]. Therefore, there needs to be additional downstream molecular events rendering the cells invasive [26].

Several studies have reported additional molecular mechanisms contributing to the progression of indolent SRC to invasive carcinoma [51, 52]. Since de-differentiated tumour cells that constitute advanced HDGC display a morphology reminiscent of mesenchymal cells, it has been proposed that EMT mediates progression from early to advanced HDGC [53]. C-Src kinase is a well-characterised inducer of EMT and has been shown to be strongly expressed in poorly differentiated and de-differentiated cells invading the muscularis mucosae [53]. In contrast, it was not expressed in intra-mucosal SRC. In line with this finding, downstream targets of c-Src, such as Fibronectin, FAK, and Stat3, were not expressed in small intra-mucosal SRC foci while immunoreactivity was seen in larger intra-mucosal SRC and de-differentiated neoplastic cells as well as advanced HDGC [54]. However, these findings were not aligned with another study which reported no differences in immunoreactivity between smaller and larger intra-mucosal SRC where immunoreactivity of cytokeratins 8/ 18 and Vimentin, which are markers of EMT, were examined by dual-label immunofluorescence [55]. Moreover, N-cadherin which is another EMT marker was not observed in majority of the intra-mucosal SRC foci [55]. Overall, these mixed findings demonstrate that the role for EMT in the development of invasive HDGC from early and indolent microscopic foci remains to be defined.

The phosphatidylinositol 3-kinase (PI3K) pathway may also play a role in the pathogenesis of HDGC. A study reported that the activated ErbB2/ ErbB3 complex in SRC binds to PI3K leading to phosphorylation of tyrosine residues and activation of downstream pathways, including p38 MAP kinase [56]. Subsequently, activation of p38 MAP kinase leads to loss of cell-cell contact by disruption of adherent junctions. Under typical conditions, MUC4 and ErbB2 are separated by tight junctions. However, they are able to interact in SRC as these junctions are absent [57]. As a result of loss of cell-cell interactions, the ErbB2/ ErbB3 signalling pathway becomes constitutively activated with uncontrolled cell proliferation contributing to malignancy progression [57]. Moreover, the MEK1 pathway enhances loss of cell-cell contact [56]. In addition, Notch signalling may be influenced by E-cadherin and mediate tumour development associated with E-cadherin deficiency since the ability to inhibit Notch-1 signalling was lost in cells transfected with mutant forms of E-cadherin. Following inhibition of Notch-1, E-cadherin-deficient cells were re-sensitised to apoptosis in a similar degree compared to wild-type E-cadherin cells [58].

In summary, although the molecular pathogenesis of HDGC has improved over the years, the exact mechanisms remain unknown [59]. It will be crucial to characterise early SRC lesions to gain greater insight into the specific signalling pathways involved in its development and progression.

## Therapeutic strategies in HDGC

In addition to PTG or endoscopic surveillance for HDGC, there are no alternate therapeutic options. Several therapeutic strategies have been evaluated in in-vitro studies in the context of pathogenic *CDH1* mutations and its downstream effects. There are 3 main therapeutic approaches which include targeting downstream signalling pathways to inhibit cell proliferation, reversing second hit mechanism of the wild-type *CDH1* allele and a synthetic lethal approach to induce cancer cell death.

As discussed previously, C-Src kinase is a well-characterised inducer of EMT which is a potential pathway mediating progression from early to advanced HDGC [53]. The upregulation of c-Src in HDGC has been linked to loss of epidermal growth factor receptor (EGFR) inhibition which is an upstream tyrosine receptor kinase of c-Src [60]. E-cadherin has an inhibitory effect on EGFR as shown by data from cell lines derived from HDGC patients with impaired extracellular domains of E-cadherin that were less able to suppress EGFR signalling [61]. Consequently, loss of EGFR inhibition increases activation of downstream components, including c-Src and PI3K [62]. In line with these findings, HDGC-derived cell lines demonstrated sensitivity to EGFR inhibition [63], suggesting it may be a potential therapeutic strategy. Furthermore, increased sensitivity to c-Src kinase inhibitor named Saracatinib and loss of viability in *CDH1*-deficient mutant cells is likely attributed to increased G-protein coupled receptor (GPCR) signalling [63]. GPCR signalling directly activates c-Src demonstrated by raised phosphatidylinositol 4,5-bisphosphate (PIP2) and phosphatidylinositol 3,4,5-trisphosphate (PIP3) levels which are second messenger intermediates.

In addition, given the high frequency of *CDH1* promoter hypermethylation, de-methylating drugs are emerging as possible therapeutic options in HDGC. Notably, *CDH1* became de-methylated and selectively re-expressed in cancer cell lines upon treatment with a histone deacetylation inhibitor named trichostatin [64], raising the possibility that such an approach may have therapeutic benefit in HDGC. Likewise, Vorinostat, a histone deacetylase inhibitor, has shown particular benefit when combined with a taxane or capecitabine and cisplatin [65]. In addition, other therapeutic options have been proposed, such as MMP-targeting agents, Hedgehog signalling and RHOA inhibition, which have been shown to improve E-cadherin cellular activities [66].

Recently, the synthetic lethality approach to screen for suitable therapeutic targets has been examined in *CDH1*-deficient cells. As loss-of-function mutations are challenging to target therapeutically, the synthetic lethal approach could be adopted in *CDH1*-deficient cells where simultaneous loss of two genes induces cell death, while inactivation of either gene alone has minimal effect on cell viability [67]. The potential of this therapeutic approach using CRISPR-Cas9 based gene editing has demonstrated promising results harnessing druggable vulnerabilities in *CDH1*-mutant cancers and profound anti-tumour effects in multiple studies as detailed in Table 1 [67–73]. To identify therapeutic leads, gene expression profiles and drug phenotypes derived from an oncology library of 1912 compounds were compared between gastric cancer cells established from a patient with metastatic HDGC harbouring c.1380delA *CDH1* germline variant and sporadic gastric cancer cells [67]. The results showed enrichment of ERK1/ ERK2 and IP3/ DAG signalling as top ranking networks in the HDGC cell line. In line with this finding, high-throughput drug screening identified several compound classes with enriched activity in HDGC cells, including mTOR, MEK, c-Src kinase, FAK and topoisomerase II inhibitors. Moreover, dual PI3K/ mTOR and topoisomerase II inhibitors demonstrated more than 100-fold increased activity in HDGC cells inducing apoptosis most effectively in cells with deficient *CDH1* function. Other drugs which have been investigated include ROS1 inhibitors [68] and Dasatinib, an inhibitor of multiple kinases [69], which preferentially induced apoptosis of E-cadherin-deficient cells. Similarly, breast and gastric cells lacking *CDH1* expression were shown to be more sensitive to allosteric AKT inhibitors than their CDH1-expressing isogenic counterparts [73]. Overall, these proof-of-concept studies demonstrate the promising potential of synthetic lethality in HDGC where pharmacological vulnerabilities selective to *CDH1*-deficient cells should be considered for molecular-targeted therapies.

**Table 1:** Proposed Models of Pathogenesis in HDGC

No.	Pathway	Downstream Targets	Proposed Mechanism	Reference
1.	Epithelial mesenchymal transition (EMT)	Fibronectin, FAK, and Stat3	Initiation phase characterised by destabilisation of adherens junctions in proliferation zone and concomitant formation of SRC	[53]
			Progression phase characterised by poor differentiation, activation of c-Src kinase and induction of EMT	
2.	ErbB	ErbB2, ErbB3, PI3K, p38 MAP kinase, Rac1, MEK1	Activated ErbB2/ ErbB3 complex binds to PI3K➔ Activation of p38 MAP kinase and adherens junctions disruption via Rac1 where MEK1 pathway enhances cell–cell dissociation	[56, 57]
3.	Notch	Notch-1, Bcl-2	• ↑ Notch-1 in E-cadherin-deficient cells➔ ↑ Bcl-2➔ ↑ apoptosis resistance	[58]

*SRC* Signet ring cell

## Outlook for future research avenues

There are several key research avenues that would benefit patients. These areas include identifying new pre-disposing genes in HDGC, elucidating molecular mechanisms that induce transition between indolent and invasive stages, and using this to evaluate novel biomarkers as well as therapeutic approaches.

**Fig. 1:**
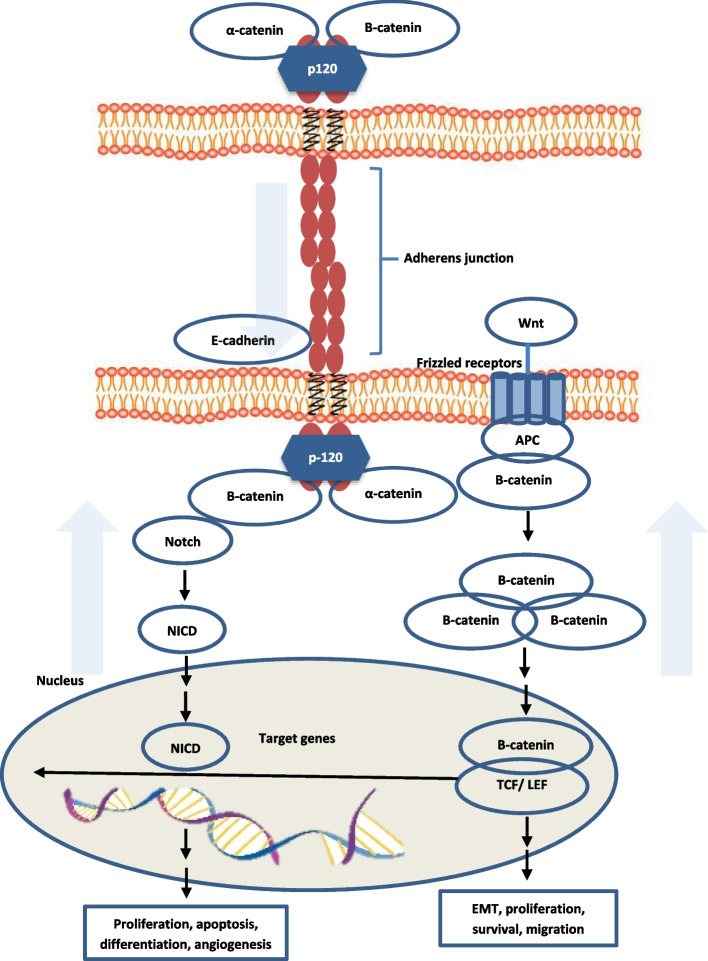
Overview of Signalling Pathways Involved in Hereditary Diffuse Gastric Cancer. Direction of arrow represent upregulation or down-regulation of pathway. NICD: Intra-cellular domain of the Notch protein; TCF/ LEF: T-cell factor/ Lymphoid enhancer factor

Multiple small SRC foci have been identified in *CDH1* germline mutation carriers. Although these foci are relatively indolent, they may progress to advanced disease. As such, there remains an urgent need to elucidate the molecular mechanisms underlying transition of indolent SRC to HDGC and identify suitable biomarkers for risk stratification of patients who are likely to progress. These mechanisms may include genetic and epigenetic triggers which shift SRC from indolent to more invasive behaviour. For example, in a study which included 12 intra-mucosal carcinomas (pT1a) and 9 widely invasive carcinomas (pT > 1, intra-mucosal SRC presented with an indolent phenotype and low proliferative index characterised by absent immunoexpression of Ki-67 and p53 while advanced carcinomas displayed an aggressive phenotype with pleomorphic cells that were immunoreactive for Ki-67 and p53 [18]. These features show that the immunohistochemical profile is different between intra-mucosal SRC and advanced HDGC providing evidence of phenotypic heterogeneity which may help provide predictive biomarkers of progression. Potential markers of progression, such as p53 and Notch, have been examined as shown in Table 2 which outlines signalling pathways involved in the pathogenesis of HDGC. A diagrammatic overview of these signalling pathways has been included in Fig. 1. Consequently, use of new targeted agents will be improved by the development of suitable biomarkers to identify patients most likely to benefit and monitor treatment efficacy.

**Table 2:** Studies on Synthetic Lethal Screen in *CDH1*-deficient Cancers

No.	Cancer Type	Study Model	Target(s)	Drug(s)	Main Findings	Reference
1.	Breast and gastric carcinoma	Breast and gastric cancer cell lines with *CDH1* knockout	ROS 1	Crizotinib and Foretinib	Induced mitotic abnormalities and multi-nucleation in E-cadherin deficient cells	[68]
2.	Mammary epithelial cell line	MCF10A and isogenic line with *CDH1* knockout	Histone deacetylase, PI3K and tyrosine kinase	Vorinostat, Entinostat	Linkages to GPCR signalling and cytoskeletal function that showed evidence of E-cadherin synthetic lethality	[70]
			PI3K	PI103		
			Tyrosine kinase	Crizotinib, Saracatinib		
3.			–	12 novel lead-like compounds	Preferentially harmed E-cadherin-deficient cells	[71]
4.			Multiple kinases (eg. BCR-ABL, DDR2, Src)	Dasatinib	Preferentially slowed growth and induced apoptosis of E-cadherin-deficient cells	[69]
5.	Mammary epithelial cell line and gastric adenocarcinoma	MCF10A and NCI-N87 isogenic lines with *CDH1* knockout	PI3K/ AKT	PI103	*CDH1*-null cells more sensitive than wild-type cells to compounds that disrupted plasma membrane composition and trafficking	[72]
			G-protein coupled receptor	Bafilomycin A1		
			Ion channels	NS3728		
			Proteosomal subunit proteins	Latrunculin B, Cytochalasin D		
			Ubiquitinylation enzymes	Ethyl-β-cyclodextrin		
6.			AKT	ARQ-092, MK2206	*CDH1* null cells were more sensitive to allosteric AKT inhibitors	[73]

*DDR2* Discoidin domain receptor tyrosine kinase 2

In particular, patient-derived organoids offer a valuable platform to elucidate molecular mechanisms of SRC progression and identify potential treatment targets as it provides a more physiological model [74, 75]. The organoid can maintain the genotype and phenotype of the original tumour [74]. Furthermore, organoids maintain the sensitivity of the original tumour to drugs making it an ideal tool for functional drug screening to identify therapeutic targets for implementation in clinical practice [76]. This model can be used not only for selection of targeted drugs but potentially extended to evaluate the role of the surrounding tumour immune microenvironment on cancer growth [77].

Loss of E-cadherin due to pathogenic *CDH1* mutations creates druggable vulnerabilities. As discussed, the synthetic lethal approach could use a targeted drug to cause cell death exclusively in tumours carrying specific genetic alterations, thereby offering a selective advantage to conventional drug approaches. Since HDGC is characterised by the absence of E-cadherin, loss of E-cadherin in early-stage DGC could be specifically targeted using a synthetic lethal approach. Chemoprevention of HDGC can be achieved by either targeting early stage SRC foci (T1a) before they have acquired the capability for invasion beyond the muscularis mucosae or by reducing the risk of epigenetic silencing of the second *CDH1* allele, leading to complete loss of E-cadherin expression and migration of epithelial cells into the lamina propria. For example, Entinostat, a histone deacetylase inhibitor, may be able to reduce cancer risk by both promoting apoptosis in established *CDH1*-null foci and restore expression of the non-mutant copy of *CDH1* in gastric organoids [77]. Of all the pan-HDAC inhibitors tested, Entinostat showed the most promising results with a synthetic lethal effect in all models [78]. The molecular vulnerability that sensitises *CDH1*-null cells to pan-HDAC inhibitors is unclear. Although the effect may be related to a specific histone or non-histone target, the maintenance of a synthetic lethal effect with multiple HDAC inhibitors suggests their effect may be more general. There are plans from the study group to investigate the use of Entinostat in combination with other potential chemoprevention drugs with a view to validate the drug in *CDH1* mutation carriers as part of a clinical trial [78]. Findings from these studies will pave the way for the development of rationally designed drug combinations for the chemoprevention of HDGC. In addition, reduction in drug dose may be enabled by the identification of synergistic combinations between synthetic lethal drugs.

Moreover, if methods could be devised to effectively deliver drugs directly to the gastric mucosa during endoscopy this would aid to minimise side effects associated with systemic therapy. Prior to undergoing PTG, *CDH1* mutation carriers typically have an extended waiting period of several months which presents a window of opportunity for treatment to be rendered. Moving forward, there is a valuable opportunity to perform a chemoprevention trial in patients who are due to undergo a gastrectomy where an endoscopic biopsy before and after therapy to assess activity against SRC lesions could be performed (Table 3).

**Table 3:** Signalling Pathways involved in Hereditary Diffuse Gastric Cancer

No.	Pathway	Function	Study Model		Main Findings	Reference
**In-vitro**						
			**No. of cell line(s)**	**No. of clinical samples**		
1.	E-cadherin/ Wnt/ EMT	Inter-cellular adhesion, cell proliferation, migration and metastasis		13	• ↓ junctional proteins • ↑ C-Src and downstream targets	[53]
2.				19	• ↑ Wnt2 and cytoplasmic/ nuclear β-catenin➔ ↑ T stage and lymph node metastasis	[79]
3.	p53	Cell cycle arrest, DNA repair, apoptosis		21	• ↑ aggressive phenotype, pleomorphic cells, Ki-67 and p53	[18]
4.	Notch	Proliferation, apoptosis, differentiation, angiogenesis	1		• ↑ Notch-1 in E-cadherin-deficient cells➔ ↑ Bcl-2➔ ↑ apoptosis resistance	[58]
**In-vivo**						
1.	Junctional-complex proteins	Inter-cellular adhesion	11 *CDH1* +/− mice		• ↓ E-cadherin (> 50% compared with normal epithelial cells) • ↓ junctional proteins	[80]

*EMT* Epithelial-mesenchymal transition

## Conclusion

In conclusion, HDGC is a rare genetic syndrome that significantly increases lifetime risk of gastric cancer with a precursor phase of SRC formation that provides a unique opportunity for early interception. There is an urgent clinical need to gain greater insight into the pathogenesis and molecular mechanisms underlying transition of indolent SRC to DGC in the context of germline *CDH1* mutations. This will facilitate risk stratification of patients who are likely to progress. Recent genomic and epigenomic profiling studies are also beginning to form an important foundation to develop targeted treatment options. Novel therapies developed based on molecular biomarkers and signalling pathways through organoid-based functional drug analysis will improve treatment options for HDGC. As loss-of-function mutations are challenging to target therapeutically, the synthetic lethal approach in *CDH1*-deficient cells has shown promising results and will need further exploration in study models. Moving forward, if we could better understand the molecular vulnerabilities there is a window of opportunity to perform a chemoprevention trial in HDGC patients who are due to undergo a gastrectomy to pave the way to improving therapy options beyond gastrectomy.

## Data Availability

Not applicable.

## Appendix

### Search strategy

A literature search was performed for relevant studies published from 2000 to 2022 examining *CDH1* germline variants in HDGC, *CDH1* second-hit mechanisms, pathogenesis of HDGC and potential therapeutic strategies. Articles were systematically searched for in scientific databases, including PubMed, ScienceDirect and Scopus. For each section, the exact search terms and combination in conjunction with results obtained have been included. Reference lists of identified studies were also screened to identify eligible additional studies. Only full-text articles written in English were included.

### Pathogenic Germline Variants in *CDH1*

**PubMed** (12/11/2022).

**Table:**

Search	Query	Hits
#4	#3 Limits: Publication Date from 2000 to 2022	119
#3	#1 AND #2	119
#2	“*CDH1* mutation or pathogenic variant or germline mutation [MeSH Major Topic]” OR “*CDH1* mutation or pathogenic variant or germline mutation [MeSH Subheading]”	1, 031
#1	“Hereditary diffuse gastric cancer [MeSH Major Topic]” OR “Hereditary diffuse gastric cancer [MeSH Subheading]”	1, 135

**ScienceDirect** (12/11/2022).

**Table:**

Search	Query	Hits
#4	#1 AND #2 AND #3 Limit: Publication Date from 2000 to 2022	45
#2	*CDH1* mutation or pathogenic variant or germline mutation	180
#1	Hereditary diffuse gastric cancer	129

**Scopus** (12/11/2022).

**Table:**

Search	Query	Hits
#2	#1 Limit: Publication Date from 2000 to 2022	202
#1	(*CDH1* mutation or pathogenic variant or germline mutation) AND hereditary diffuse gastric cancer	202

### Second-hit Mechanism of *CDH1* Wild-type Allele

**PubMed** (15/11/2022).

**Table:**

Search	Query	Hits
#4	#3 Limits: Publication Date from 2000 to 2022	4
#3	#1 AND #2	4
#2	“Hereditary diffuse gastric cancer [MeSH Major Topic]” OR “Hereditary diffuse gastric cancer [MeSH Subheading]”	1, 135
#1	“Second-hit mechanism [MeSH Major Topic] OR “Second-hit mechanism [MeSH Subheading]”	3, 342

**ScienceDirect** (15/11/2022).

**Table:**

Search	Query	Hits
#2	#1 Limit: Publication Date from 2000 to 2022	2
#1	Second-hit mechanism AND hereditary diffuse gastric cancer	2

**Scopus** (15/11/2022).

**Table:**

Search	Query	Hits
#2	#1 Limit: Publication Date from 2000 to 2022	3
#1	Second-hit mechanism AND hereditary diffuse gastric cancer	3

### Proposed Models of Pathogenesis in HDGC

**PubMed** (16/11/2022).

**Table:**

Search	Query	Hits
#4	#3 Limits: Publication Date from 2000 to 2022	10
#3	#1 AND #2	10
#2	“Hereditary diffuse gastric cancer [MeSH Major Topic]” OR “Hereditary diffuse gastric cancer [MeSH Subheading]”	1, 135
#1	“Pathogenesis or development or progression [MeSH Terms]”	354, 479

**ScienceDirect** (16/11/2022).

**Table:**

Search	Query	Hits
#2	#1 Limit: Publication Date from 2000 to 2022	4
#1	(Pathogenesis or development or progression) AND hereditary diffuse gastric cancer	4

**Scopus** (16/11/2022).

**Table:**

Search	Query	Hits
#2	#1 Limit: Publication Date from 2000 to 2022	70
#1	(Pathogenesis or development or progression) AND hereditary diffuse gastric cancer	70

### Therapeutic Strategies in HDGC

**PubMed** (18/11/2022).

**Table:**

Search	Query	Hits
#4	#3 Limits: Publication Date from 2000 to 2022	8
#3	#1 AND #2	4
#2	“Hereditary diffuse gastric cancer [MeSH Major Topic]” OR “Hereditary diffuse gastric cancer [MeSH Subheading]”	1, 135
#1	“Therapy or therapeutic strategy or treatment [MeSH Major Topic] OR “Second-hit mechanism [MeSH Subheading]” OR “Therapy or therapeutic strategy or treatment [MeSH Term]”	649, 721

**ScienceDirect** (18/11/2022).

**Table:**

Search	Query	Hits
#2	#1 Limit: Publication Date from 2000 to 2022	0
#1	Therapy or therapeutic strategy or treatment AND hereditary diffuse gastric cancer	0

**Scopus** (18/11/2022).

**Table:**

Search	Query	Hits
#2	#1 Limit: Publication Date from 2000 to 2022	0
#1	Therapy or therapeutic strategy or treatment AND hereditary diffuse gastric cancer	0

### Outlook for Future Research Avenues

**PubMed** (18/11/2022).

**Table:**

Search	Query	Hits
#5	(#1 OR #2) AND #3 AND #4 Limits: Publication Date from 2000 to 2022	6
#4	(#1 OR #2) AND #3 AND #4	10
#3	“pathogenesis or development OR “progression”{MeSH Term]	14,371,763
#2	“hereditary gastric cancer or malignan* or tumour or neoplasm”[MeSH Major Topic]	1, 356
#1	“signalling pathway”[MeSH Major Topic] OR “signalling pathway”[MeSH Subheading]	337, 725

**ScienceDirect** (18/11/2022).

**Table:**

Search	Query	Hits
#2	#1 Limit: Publication Date from 2000 to 2022	4
#1	“Upregulated signalling pathway or signalling pathway or pathway” AND “hereditary gastric cancer or malignan* or tumour or neoplasm” AND “pathogenesis or development or progression”	4

**Scopus** (18/11/2022).

**Table:**

Search	Query	Hits
#2	#1 Limit: Publication Date from 2000 to 2022	5
#1	“Upregulated signalling pathway or signalling pathway or pathway” AND “hereditary gastric cancer or malignan* or tumour or neoplasm” AND “pathogenesis or development or progression”	6

## References

[1] Blair V, Martin I, Shaw D, Winship I, Kerr D, Arnold J (2006). Hereditary diffuse gastric cancer: diagnosis and management. Clin Gastroenterol Hepatol.

[2] Carneiro F, Huntsman DG, Smyrk TC, Owen DA, Seruca R, Pharoah P (2004). Model of the early development of diffuse gastric cancer in E-cadherin mutation carriers and its implications for patient screening. J Pathol.

[3] Bryant DM, Stow JL (2004). The ins and outs of E-cadherin trafficking. Trends Cell Biol.

[4] Lecuit T, Yap AS (2015). E-cadherin junctions as active mechanical integrators in tissue dynamics. Nat Cell Biol.

[5] Mendonsa AM, Na TY, Gumbiner BM (2018). E-cadherin in contact inhibition and cancer. Oncogene.

[6] Huber AH, Weis WI (2001). The structure of the beta-catenin/E-cadherin complex and the molecular basis of diverse ligand recognition by beta-catenin. Cell.

[7] Pećina-Slaus N (2003). Tumor suppressor gene E-cadherin and its role in normal and malignant cells. Cancer Cell Int.

[8] Liu X, Chu KM. E-cadherin and gastric cancer: cause, consequence, and applications. Biomed Res Int. 2014:637308. 10.1155/2014/637308 PMC4145387 25184143

[9] Fitzgerald RC, Caldas C (2004). Clinical implications of E-cadherin associated hereditary diffuse gastric cancer. Gut.

[10] Corso G, Pedrazzani C, Pinheiro H, Fernandes E, Marrelli D, Rinnovati A (2011). E-cadherin genetic screening and clinico-pathologic characteristics of early onset gastric cancer. Eur J Cancer.

[11] Guilford P, Hopkins J, Harraway J (1998). E-cadherin germline mutations in familial gastric cancer. Nature.

[12] Schrader KA, Masciari S, Boyd N, Wiyrick S, Kaurah P, Senz J (2008). Hereditary diffuse gastric cancer: association with lobular breast cancer. Familial Cancer.

[13] Lerner BA, Llor X (2020). Genetic gastric Cancer risk syndromes. Curr Treat Options Gastroenterol.

[14] Blair VR, McLeod M, Carneiro F, Coit DG, D'Addario JL, van Dieren JM (2020). Hereditary diffuse gastric cancer: updated clinical practice guidelines. Lancet Oncol.

[15] van der Kaaij RT, van Kessel JP, van Dieren JM, Snaebjornsson P, Balagué O, van Coevorden F (2018). Outcomes after prophylactic gastrectomy for hereditary diffuse gastric cancer. Br J Surg.

[16] Li J, Zhang Y, Hu DM, Gong TP, Xu R, Gao J (2020). Impact of postoperative complications on long-term outcomes of patients following surgery for gastric cancer: a systematic review and meta-analysis of 64 follow-up studies. Asian J Surg.

[17] Monster JL, Kemp LJS, Gloerich M, van der Post RS (2022). Diffuse gastric cancer: emerging mechanisms of tumor initiation and progression. Biochim Biophys Acta Rev Cancer.

[18] van der Post RS, Gullo I, Oliveira C, Tang LH, Grabsch HI, O'Donovan M (2016). Histopathological, molecular, and genetic profile of hereditary diffuse gastric Cancer: current knowledge and challenges for the future. Adv Exp Med Biol.

[19] Humar B, Toro T, Graziano F, Muller H, Dobbie Z, Kwang-Yang H, Eng C, Hampel H, Gilbert D, Winship I, Parry S, Ward R, Findlay M, Christian A, Tucker M, Tucker K, Merriman T, Guilford P (2002). Novel germline CDH1 mutations in hereditary diffuse gastric cancer families. Hum Mutat.

[20] Kaurah P, MacMillan A, Boyd N, Senz J, De Luca A, Chun N (2007). Founder and recurrent CDH1 mutations in families with hereditary diffuse gastric cancer. JAMA.

[21] Brooks-Wilson AR, Kaurah P, Suriano G, Leach S, Senz J, Grehan N (2004). D. Germline E-cadherin mutations in hereditary diffuse gastric cancer: assessment of 42 new families and review of genetic screening criteria. J Med Genet.

[22] Guilford P, Humar B, Blair V (2010). Hereditary diffuse gastric cancer: translation of CDH1 germline mutations into clinical practice. Gastric Cancer.

[23] Bacani JT, Soares M, Zwingerman R, di Nicola N, Senz J, Riddell R (2006). CDH1/E-cadherin germline mutations in early-onset gastric cancer. J Med Genet.

[24] Oliveira C, Senz J, Kaurah P, Pinheiro H, Sanges R, Haegert A (2009). Germline CDH1 deletions in hereditary diffuse gastric cancer families. Hum Mol Genet.

[25] van der Post RS, Vogelaar IP, Carneiro F, Guilford P, Huntsman D, Hoogerbrugge N (2015). Hereditary diffuse gastric cancer: updated clinical guidelines with an emphasis on germline CDH1 mutation carriers. J Med Genet.

[26] Luo W, Fedda F, Lynch P, Tan D (2018). CDH1 gene and Hereditary diffuse gastric Cancer syndrome: molecular and histological alterations and implications for diagnosis and treatment. Front Pharmacol.

[27] Hakkaart C, Ellison-Loschmann L, Day R, Sporle A, Koea J, Harawira P (2019). Germline CDH1 mutations are a significant contributor to the high frequency of early-onset diffuse gastric cancer cases in New Zealand Māori. Familial Cancer.

[28] Lo W, Zhu B, Sabesan A, Wu HH, Powers A, Sorber RA (2019). Associations of CDH1 germline variant location and cancer phenotype in families with hereditary diffuse gastric cancer (HDGC). J Med Genet.

[29] Melo S, Figueiredo J, Fernandes MS, Gonçalves M, Morais-de-Sá E, Sanches JM, Seruca R (2017). Predicting the functional impact of CDH1 missense mutations in hereditary diffuse gastric Cancer. Int J Mol Sci.

[30] Suriano G, Oliveira C, Ferreira P, Machado JC, Bordin M, De Wever O (2003). Identification of CDH1 germline missense mutations associated with functional inactivation of the E-cadherin protein in young gastric cancer probands. Hum Mol Genet.

[31] Kluijt I, Siemerink EJ, Ausems MG, van Os TA, de Jong D, Simoes-Correia (2012). CDH1-related hereditary diffuse gastric cancer syndrome: clinical variations and implications for counseling. Int J Cancer.

[32] Wang HD, Ren J, Zhang L (2004). CDH1 germline mutation in hereditary gastric carcinoma. World J Gastroenterol.

[33] Graziano F, Humar B, Guilford P (2003). The role of the E-cadherin gene (CDH1) in diffuse gastric cancer susceptibility: from the laboratory to clinical practice. Ann Oncol.

[34] Clark DF, Michalski ST, Tondon R, Nehoray B, Ebrahimzadeh J, Hughes SK (2020). Loss-of-function variants in CTNNA1 detected on multigene panel testing in individuals with gastric or breast cancer. Genet Med.

[35] Huiping C, Kristjansdottir S, Jonasson JG, Magnusson J, Egilsson V, Ingvarsson S (2001). Alterations of E-cadherin and beta-catenin in gastric cancer. BMC Cancer.

[36] Schuetz JM, Leach S, Kaurah P, Jeyes J, Butterfield Y, Huntsman D, Brooks-Wilson AR (2012). Catenin family genes are not commonly mutated in hereditary diffuse gastric cancer. Cancer Epidemiol Biomark Prev.

[37] Carneiro F (2022). Familial and hereditary gastric cancer, an overview. Best Pract Res Clin Gastroenterol.

[38] Jakubowska A, Nej K, Huzarski T, Scott RJ, Lubiński J (2002). BRCA2 gene mutations in families with aggregations of breast and stomach cancers. Br J Cancer.

[39] Hansford S, Kaurah P, Li-Chang H, Woo M, Senz J, Pinheiro H, et al. Hereditary diffuse gastric cancer syndrome: CDH1 mutations and beyond. JAMA Oncol. 2015. 10.1001/jamaoncol.2014.168 26182300

[40] Ansari S, Gantuya B, Tuan VP, Yamaoka Y (2018). Diffuse gastric Cancer: a summary of analogous contributing factors for its molecular pathogenicity. Int J Mol Sci.

[41] Fewings E, Larionov A, Redman J, Goldgraben MA, Scarth J, Richardson S (2018). Germline pathogenic variants in PALB2 and other cancer-predisposing genes in families with hereditary diffuse gastric cancer without CDH1 mutation: a whole-exome sequencing study. Lancet Gastroenterol Hepatol.

[42] Fitzgerald RC, Hardwick R, Huntsman D, Carneiro F, Guilford P, Blair V (2010). Hereditary diffuse gastric cancer: updated consensus guidelines for clinical management and directions for future research. J Med Genet.

[43] Cosma LS, Schlosser S, Tews HC, Müller M, Kandulski A (2022). Hereditary diffuse gastric Cancer: molecular genetics, biological mechanisms and current therapeutic approaches. Int J Mol Sci.

[44] Oliveira C, Sousa S, Pinheiro H, Karam R, Bordeira-Carriço R, Senz J (2009). Quantification of epigenetic and genetic 2nd hits in CDH1 during hereditary diffuse gastric cancer syndrome progression. Gastroenterology.

[45] Barber M, Murrell A, Ito Y, Maia AT, Hyland S, Oliveira C (2008). Mechanisms and sequelae of E-cadherin silencing in hereditary diffuse gastric cancer. J Pathol.

[46] Oliveira C, de Bruin J, Nabais S, Ligtenberg M, Moutinho C, Nagengast FM (2004). Intragenic deletion of CDH1 as the inactivating mechanism of the wild-type allele in an HDGC tumour. Oncogene.

[47] Grady WM, Willis J, Guilford PJ, Dunbier AK, Toro TT, Lynch H (2000). Methylation of the CDH1 promoter as the second genetic hit in hereditary diffuse gastric cancer. Nat Genet.

[48] Guilford P, Blair V, More H, Humar B (2007). A short guide to hereditary diffuse gastric cancer. Hered Cancer Clin Pract.

[49] Kiso M, Urabe Y, Ito M, Masuda K, Boda T, Kotachi T (2020). Clinical and genomic characteristics of mucosal signet-ring cell carcinoma in helicobacter pylori-uninfected stomach. BMC Gastroenterol.

[50] Mimata A, Fukamachi H, Eishi Y, Yuasa Y (2011). Loss of E-cadherin in mouse gastric epithelial cells induces signet ring-like cells, a possible precursor lesion of diffuse gastric cancer. Cancer Sci.

[51] Mi EZ, di Pietro M, O'Donovan M, Hardwick RH, Richardson S, Ziauddeen H (2018). Comparative study of endoscopic surveillance in hereditary diffuse gastric cancer according to CDH1 mutation status. Gastrointest Endosc.

[52] Gullo I, Devezas V, Baptista M, Garrido L, Castedo S, Morais R (2018). Phenotypic heterogeneity of hereditary diffuse gastric cancer: report of a family with early-onset disease. Gastrointest Endosc.

[53] Humar B, Fukuzawa R, Blair V, Dunbier A, More H, Charlton A (2007). Destabilized adhesion in the gastric proliferative zone and c-Src kinase activation mark the development of early diffuse gastric cancer. Cancer Res.

[54] Guarino M (2010). Src signaling in cancer invasion. J Cell Physiol.

[55] Barber ME, Save V, Carneiro F, Dwerryhouse S, Lao-Sirieix P, Hardwick RH, Caldas C, Fitzgerald RC (2008). Histopathological and molecular analysis of gastrectomy specimens from hereditary diffuse gastric cancer patients has implications for endoscopic surveillance of individuals at risk. J Pathol.

[56] Xu Q, Karouji Y, Kobayashi M, Ihara S, Konishi H, Fukui Y (2003). The PI 3-kinase-Rac-p38 MAP kinase pathway is involved in the formation of signet-ring cell carcinoma. Oncogene.

[57] Yokoyama A, Shi BH, Kawai T, Konishi H, Andoh R, Tachikawa H, Ihara S, Fukui Y (2007). Muc4 is required for activation of ErbB2 in signet ring carcinoma cell lines. Biochem Biophys Res Commun.

[58] Ferreira AC, Suriano G, Mendes N, Gomes B, Wen X, Carneiro F, Seruca R, Machado JC (2012). E-cadherin impairment increases cell survival through notch-dependent upregulation of Bcl-2. Hum Mol Genet.

[59] Okoshi R, Shu CL, Ihara S, Fukui Y (2013). Scattering of MCF7 cells by heregulin ß-1 depends on the MEK and p38 MAP kinase pathway. PLoS One.

[60] Qian X, Karpova T, Sheppard AM, McNally J, Lowy DR (2014). E-cadherin-mediated adhesion inhibits ligand-dependent activation of diverse receptor tyrosine kinases. EMBO J.

[61] Mateus AR, Seruca R, Machado JC, Keller G, Oliveira MJ, Suriano G (2007). EGFR regulates RhoA-GTP dependent cell motility in E-cadherin mutant cells. Hum Mol Genet.

[62] Belli S, Esposito D, Servetto A, Pesapane A, Formisano L, Bianco R (2020). C-Src and EGFR inhibition in molecular Cancer therapy: what Else can we improve?. Cancers (Basel).

[63] Li D, Lo W, Rudloff U (2018). Merging perspectives: genotype-directed molecular therapy for hereditary diffuse gastric cancer (HDGC) and E-cadherin-EGFR crosstalk. Clin Transl Med.

[64] Li Y, Seto E (2016). HDACs and HDAC inhibitors in Cancer development and therapy. Cold Spring Harb Perspect Med.

[65] Yoo C, Ryu MH, Na YS, Ryoo BY, Lee CW, Kang YK (2016). Vorinostat in combination with capecitabine plus cisplatin as a first-line chemotherapy for patients with metastatic or unresectable gastric cancer: phase II study and biomarker analysis. Br J Cancer.

[66] Carneiro P, Figueiredo J, Bordeira-Carriço R, Fernandes MS, Carvalho J, Oliveira C, Seruca R (2013). Therapeutic targets associated to E-cadherin dysfunction in gastric cancer. Expert Opin Ther Targets.

[67] Chen I, Mathews-Greiner L, Li D, Abisoye-Ogunniyan A, Ray S, Bian Y, Shukla V, Zhang X, Guha R, Thomas C, Gryder B, Zacharia A, Beane JD, Ravichandran S, Ferrer M, Rudloff U (2017). Transcriptomic profiling and quantitative high-throughput (qHTS) drug screening of CDH1 deficient hereditary diffuse gastric cancer (HDGC) cells identify treatment leads for familial gastric cancer. J Transl Med.

[68] Bajrami I, Marlow R, van de Ven M, Brough R, Pemberton HN, Frankum J (2018). E-cadherin/ROS1 inhibitor synthetic lethality in breast Cancer. Cancer Discov.

[69] Bougen-Zhukov N, Decourtye-Espiard L, Mitchell W, Redpath K, Perkinson J, Godwin T, Black MA, Guilford P (2022). E-cadherin-deficient cells are sensitive to the multikinase inhibitor Dasatinib. Cancers (Basel).

[70] Telford BJ, Chen A, Beetham H, Frick J, Brew TP, Gould CM (2015). Synthetic lethal screens identify vulnerabilities in GPCR signaling and cytoskeletal organization in E-cadherin-deficient cells. Mol Cancer Ther.

[71] Beetham H, Chen A, Telford BJ, Single A, Jarman KE, Lackovic K, Luxenburger A, Guilford P (2019). A high-throughput screen to identify novel synthetic lethal compounds for the treatment of E-cadherin-deficient cells. Sci Rep.

[72] Godwin TD, Kelly ST, Brew TP, Bougen-Zhukov NM, Single AB, Chen A (2019). E-cadherin-deficient cells have synthetic lethal vulnerabilities in plasma membrane organisation, dynamics and function. Gastric Cancer.

[73] Bougen-Zhukov N, Nouri Y, Godwin T, Taylor M, Hakkaart C, Single A (2019). Allosteric AKT inhibitors target synthetic lethal vulnerabilities in E-cadherin-deficient cells. Cancers (Basel).

[74] Xu H, Jiao D, Liu A, Wu K (2022). Tumor organoids: applications in cancer modeling and potentials in precision medicine. J Hematol Oncol.

[75] Verduin M, Hoeben A, De Ruysscher D, Vooijs M (2021). Patient-derived Cancer organoids as predictors of treatment response. Front Oncol.

[76] Vlachogiannis G, Hedayat S, Vatsiou A, Jamin Y, Fernández-Mateos J, Khan K (2018). Patient-derived organoids model treatment response of metastatic gastrointestinal cancers. Science.

[77] Neal JT, Li X, Zhu J, Giangarra V, Grzeskowiak CL, Ju J (2018). Organoid modeling of the tumor immune microenvironment. Cell.

[78] Decourtye-Espiard L, Bougen-Zhukov N, Godwin T, Brew T, Schulpen E, Black MA, Guilford P (2021). E-cadherin-deficient epithelial cells are sensitive to HDAC inhibitors. Cancers (Basel).

[79] Cheng XX, Wang ZC, Chen XY, Sun Y, Kong QY, Liu J, Li H (2005). Correlation of Wnt-2 expression and beta-catenin intracellular accumulation in Chinese gastric cancers: relevance with tumour dissemination. Cancer Lett.

[80] Humar B, Blair V, Charlton A, More H, Martin I, Guilford P (2009). E-cadherin deficiency initiates gastric signet-ring cell carcinoma in mice and man. Cancer Res.

